# High tibial osteotomy in Sweden, 1998–2007: A population-based study of the use and rate of revision to knee arthroplasty

**DOI:** 10.3109/17453674.2012.688725

**Published:** 2012-06-04

**Authors:** Annette W-Dahl, Otto Robertsson, L Stefan Lohmander

**Affiliations:** ^1^Department of Orthopedics, Clinical Sciences Lund, Lund University, Lund; ^2^Department of Orthopedics, Skåne University Hospital Lund, Lund, Sweden; ^3^Research Unit for Musculoskeletal Function and Physiotherapy; ^4^Department of Orthopedics and Traumatology, University of Southern Denmark, Odense, Denmark; Correspondence: annette.w-dahl@med.lu.se

## Abstract

**Background and purpose:**

Most studies on high tibial osteotomies (HTOs) have been hospital-based and have included a limited number of patients. We evaluated the use and outcome—expressed as rate of revision to knee arthroplasty—of HTO performed in Sweden with 9 million inhabitants, 1998–2007.

**Patients and methods:**

3, 161 HTO procedures on patients 30 years or older (69% men) who were operated on for knee osteoarthritis in Sweden, 1998–2007, were identified through the inpatient and outpatient care registers of the Swedish National Board of Health and Welfare. Pertinent data were verified through surgical records. Conversions of HTO to knee arthroplasty before 2010 were identified through the Swedish Knee Arthroplasty Register (SKAR). The 10-year survival was determined using revision to an arthroplasty as the endpoint.

**Results:**

The number of HTOs decreased by one third between 1998 and 2007, from 388 operations a year to 257 a year. Most of the HTOs were performed with open wedge osteotomy using external fixation. The cumulative revision rate at 10 years was 30% (95% CI: 28–32). The risk of revision increased with increasing age and was higher in women than in men (RR = 1.3, CI: 1.1–1.5).

**Interpretation:**

If being without an artificial joint implant is considered to be beneficial, then HTO is an excellent alternative to knee arthroplasty in younger and/or physically active patients suffering from knee osteoarthritis.

Among the surgical options for treatment of knee osteoarthritis (OA), high tibial osteotomy (HTO) is a joint-preserving alternative which is most commonly used in younger and/or physically active patients.

HTO can be performed with different methods, such as closed wedge osteotomy, open wedge osteotomy, and dome-shaped osteotomy. It has been used as a surgical treatment for OA since the 1950s, and has served as a standard treatment for unicompartmental knee OA. However, the use of HTO has decreased during the last 3 decades, concomitant with increasing use of knee arthroplasty.

At the beginning of the 1980s, HTO was estimated to constitute about 30% of the primary knee reconstruction surgery in Sweden (Tjörnstrand et al. 1981), decreasing to about 20% during the period 1989–1991 (Knutson et al. 1994). Information from the national database on inpatient procedures on the number of patients operated on by angle, rotation, or correction osteotomy in the knee or tibia from 1998–2007 showed a further decline by about 40% (W-Dahl et al. 2010a).

Many follow-up studies on HTOs have reported good to excellent clinical results in the majority of patients with declining results over time and an increasing risk of revision after 10–15 years (Odenbring et al. 1990, Coventry et al. 1993, Naudie et al. 1999, Aglietti et al. 2003, Sprenger and Doerzbacher 2003, Koshino et al. 2004, Flecher et al. 2006, Papachristou et al. 2006, Akizuki et al. 2008, Gstottner et al. 2008, van Raaij et al. 2008, W-Dahl et al. 2010a, Schallberger et al. 2011). There have been very few publications involving more than 100 patients, and they have shown rates of revision at 10 years of between 5% and 20% (Odenbring et al. 1990, Flecher et al. 2006, Gstottner et al. 2008, W-Dahl et al. 2010a).

In contrast to knee arthroplasties, we have found no national registrations of HTOs, and our knowledge of its use—including methods, techniques, patients, and outcome—is incomplete.

In this study we investigated the use of HTO in Sweden during the period 1998–2007, and also the outcome expressed as rate of conversion to knee arthroplasty.

## Methods

The Swedish National Board of Health and Welfare patient register (PAS) contains the patient’s personal identification number (including information on date of birth and sex), admission date, discharge date, surgical code, diagnosis code (ICD-10), and operating hospital. Information on patients operated on with the surgical code NGK59 (angle, rotation, or correction osteotomy in the knee or tibia) in combination with the ICD-10 code M17 (knee osteoarthritis) during the years 1998–2007 was gathered from the PAS.

For each of the 74 hospitals that were identified as having performed HTOs during 1998–2007, each orthopedic department was asked to supply medical records of each of the relevant patients in order to identify the side operated, and to verify the indication for surgery, diagnosis, and surgical date.

As the PAS has registration based on admission, simultaneous bilateral surgeries become registered as one admission. Such operations, not included in the PAS but identified through information from the medical records, were added. When the operating unit showed disagreement with the PAS regarding the diagnosis being OA or the surgery being an HTO, the patient was excluded. Further re-osteotomies and patients younger than 30 years were excluded.

Information from the Swedish Knee Arthroplasty Register (SKAR) was used to calculate the proportion of HTOs out of a total including unicompartmental and total knee arthroplasties (UKA and TKA). Furthermore, it was checked how many of the HTO’s had been converted to knee arthroplasty before 2011. The Swedish Knee Arthroplasty Register (SKAR) was started in 1975, and all hospitals performing knee arthroplasties in Sweden report both the primary procedure and the revision procedures to the SKAR. The coverage of the SKAR is 100% and the completeness is 97% (SKAR 2010.)

If it was not possible to identify the HTO side from the medical records (6 procedures) and the patient had later undergone knee arthroplasty, we assumed a worst-case scenario of the case being a conversion of the HTO.

The study was approved by the Ethics Committee of the Medical Faculty, Lund University (88/2008), and was performed in accordance with the Declaration of Helsinki.

### Statistics

Cumulative revision rate (CRR) curves were produced using the life table method with monthly intervals. The 95% confidence intervals (CIs) were calculated using the Wilson quadratic equation with Greenwood and Peto effective sample-size estimates (Dorey et al. 1993). When comparing the risk of age groups and sex, Cox regression was used and relative risk estimates (RR) with CI. Adjustment was made for sex, year of surgery, and age category (30–39, 40–49, 50–59, and 60+ years). In common with many other registry-based studies, BMI and grade of OA were not included in the adjustments. Bilateral observations were included in the data analyzed, but without consideration of subject dependency, as this was not found to be an issue when estimating CRR after knee arthroplasty (Robertsson and Ranstam 2003). Statistical analyses were carried out using Stata version 11.

## Results

**Figure 1.**
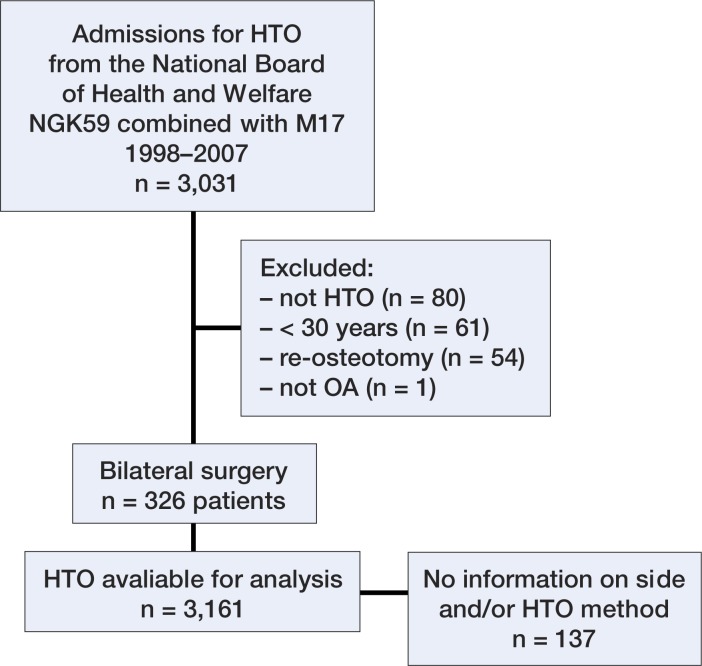
Flow chart of the study.

3,161 operations in 2,835 patients who met the criteria of primary HTO (NGK59) in combination with knee OA (M17) and who were 30 years of age or older in the period 1998–2007 were identified through the Swedish National Board of Health and Welfare (Figure 1). The mean age at osteotomy was 52 years (SD 7.5); 69% of the patients were males. 98% were 65 years of age or younger, the majority (71%) of them being younger than 55. The median follow-up time was 8.7 (3–13) years. By reviewing the medical records of the patients identified in the PAS, the diagnosis and procedure combined was verified as being correct in 94% of the procedures.

**Figure 2.**
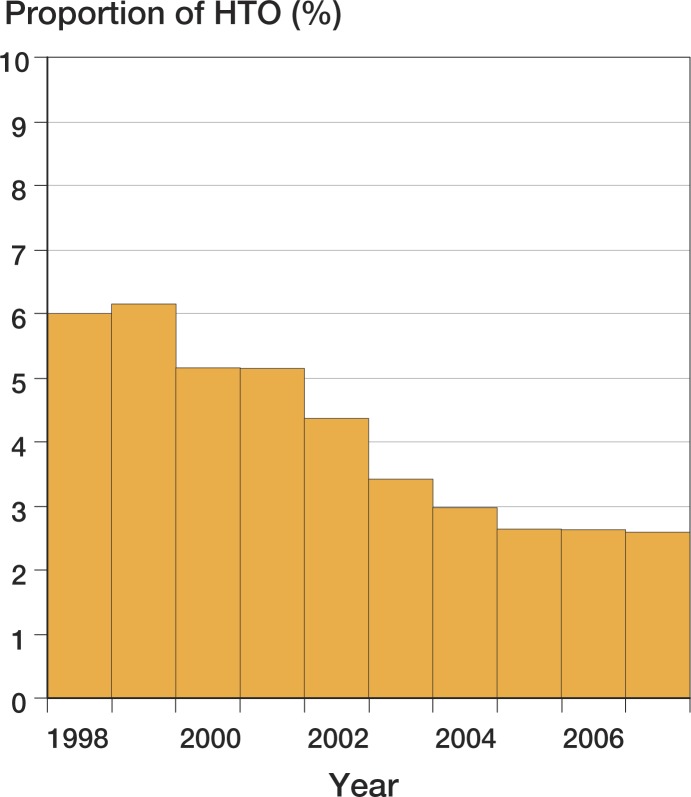
HTO as proportion of primary knee reconstruction surgery in Sweden, 1998–2007.

In absolute numbers, high tibial osteotomies decreased 34% between the years 1998 and 2007, from 388 operations to 257 per year. In 1998, HTO constituted 6.8% of the primary knee reconstruction surgery in Sweden, as compared to 2.5% in 2007 (Figure 2). Less than half of the hospitals (35/74) performed HTO surgery each year during the 10-year period, and 36% of the HTOs were performed in only 7 hospitals. In 1998, 60% of the HTOs were performed in clinics that performed less than 15 HTOs, while the corresponding figure in 2007 was 70%.

**Figure 3.**
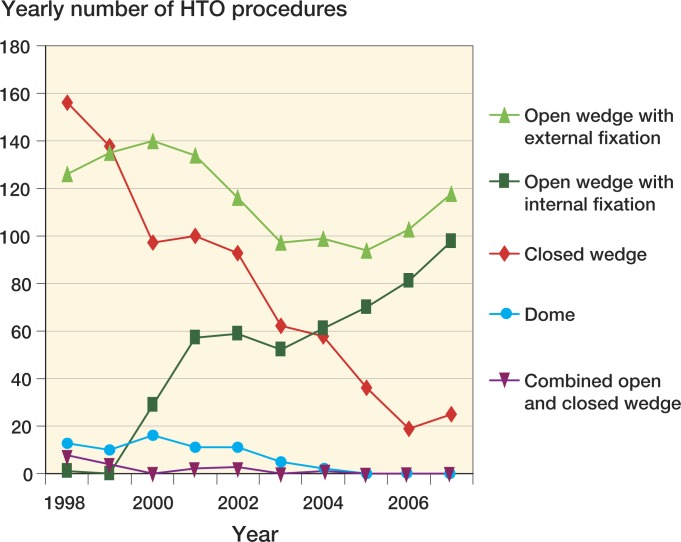
The frequency of the different methods of HTO per year.

The use of open-wedge osteotomy with internal fixation started in the year 2000. Open-wedge osteotomy with external fixation was the most common procedure used during 1998–2007, followed by closed-wedge osteotomy (Figure 3). The use of closed-wedge osteotomy decreased, while the use of open wedge osteotomy with internal or external fixation increased (Figure 3).

**Figure 4.**
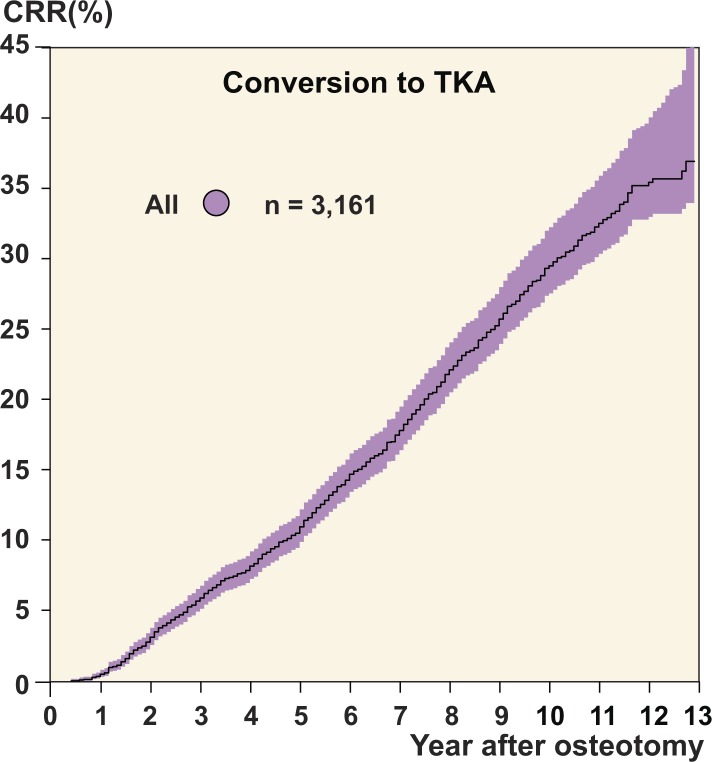
Cumulative revision rate (CRR) of HTO, with follow-up to 2010.

The 10-year risk of an HTO being converted to an arthroplasty was 30% (CI: 28–32) and the 13-year risk was 37% (CI: 34–57) (Figure 4). 730 HTOs were later converted to an arthroplasty. 6 additional cases were considered to be conversions, in a worst-case scenario whereby the HTO side was unknown and the patient had undergone a later knee arthroplasty.

**Figure 5.**
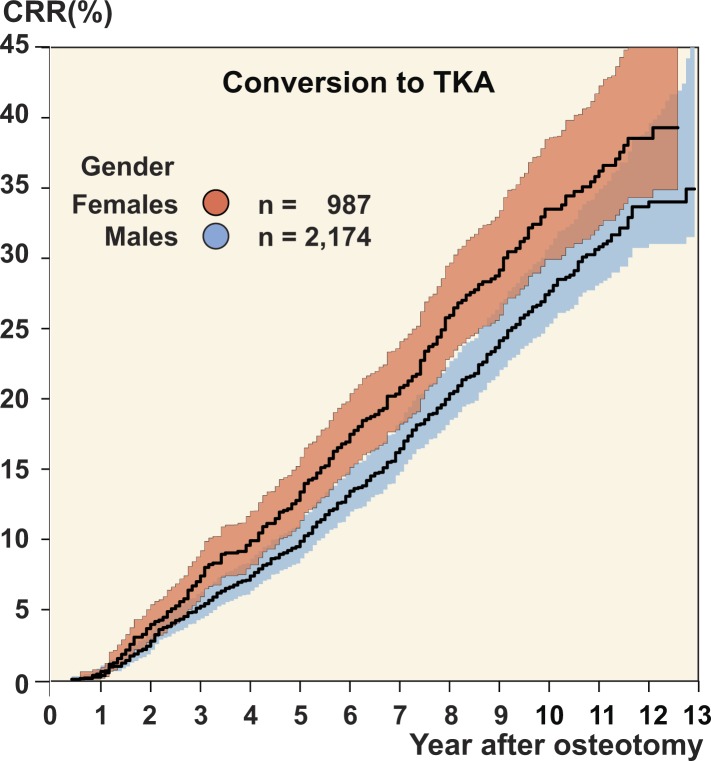
Cumulative revision rate (CRR) in men and women.

Most of the conversions were to a TKA (36 were to a UKA and 700 were to a TKA) (Table). Using Cox regression, the risk of revision, after adjusting for age and year of surgery, was found to be higher in women than in men (Figure 5), with a risk ratio (RR) of 1.3 (95% CI: 1.1–1.5). Furthermore, the risk of revision increased with increasing age. Compared to the youngest age group (30–39 years), the risk of revision increased in the older age groups, with RR = 2 (CI: 1.3–3.3) in the 40- to 49-year age group, RR = 2.7 (CI: 1.8–4.1) in the 50- to 59-year age group, and RR = 2.5 (CI: 1.6–4) in patients who were 60 years old or more.

**Table:** Frequency of type of prosthesis used for the high tibial osteotomies that were converted to knee arthroplasty

Type	n = 736
TKA without patella	651
TKA with patella	49
UKA medial	35
UKA lateral	1

TKA: total knee arthroplasty; UKA: unicompartmental knee arthroplasty.

## Discussion

This population-based retrospective study on HTOs in a whole nation over a 10-year period showed a decrease in the use of HTO in OA patients over the study period. For high tibial osteotomies performed during 1998–2007 in Sweden, we found that they were most common in men and that they were used almost exclusively for patients less than 65 years of age.

The CRR of 30% indicates that 70% of the HTOs had not been converted at 10 years after surgery. To our knowledge, this long-term outcome study of 3,161 HTOs is the largest of its kind. Furthermore, it was population-based—covering a whole nation—with 96% of the procedures identified through the administrative database and verified in medical records by diagnosis and procedure.

We used the surgical and diagnosis code to identify the patients, and we assumed that the correct codes had been registered. It is likely that some HTOs were incorrectly coded and thus missed. However, we feel confident that the absolute majority of surgeries were captured, and that bias due to missing cases or loss to follow-up was minimal. 137 procedures were not identified in the clinics, where the HTO had been registered as having been performed in the administrative PAS register. These procedures may have been misclassified by diagnosis, surgical code, or operating hospital. The outpatient care register started in 2002, so outpatient care surgery performed before that year was not available. However, the number of HTOs performed in outpatient care before 2002 was low. Registration in the PAS register is the basis of payment in the Swedish healthcare system, and this ensures that there is a low rate of missing information from both the public and the private sector.

At the national level, the proportion of HTOs decreased during the period 1998–2007, as compared to UKA and TKA which is consistent with earlier reports (Tjörnstrand et al. 1981, Knutson et al. 1994, W-Dahl et al. 2010a). For 2007, we found that HTOs represented less than 3% of the primary knee reconstruction surgeries.

During the past decade, the numbers of knee arthroplasty procedures has more than doubled in Sweden (SKAR 2010). Of patients younger than 55 years, who represent the majority of osteotomy patients, the incidence of UKA has doubled and that of TKA has quintupled (W-Dahl et al. 2010a). However, in spite of the decreasing use of HTO, it was used more frequently than UKA at the end of the observation period (W-Dahl et al. 2010).

The “industrialization” of knee arthroplasty, confining surgery to high-volume units (W-Dahl et al. 2010a), together with economic incitements for knee replacement, may have contributed to the reduced use of HTO. Most of the osteotomies studied were done in clinics performing less than 15 operations a year. For UKA, it has been shown that hospitals performing less than 23 UKAs a year had a 1.6 times higher revision rate than units that performed more (Robertsson et al. 2003). It is probable that similar factors influence outcome in HTO, suggesting that there is a need to concentrate HTO surgery to fewer units.

Our population-based study of 3,161 HTOs showed that both increasing age and female sex were associated with an increased risk of conversion to knee replacement. Previous reports on smaller cohorts have been inconsistent, showing either an increased revision risk with age (Naudie et al 1999, Fletcher et al 2006, Trieb et al. 2006, Gstottner et al. 2008) or the converse (Odenbring et al. 1990, Sprenger and Doerzbacher 2003, Spahn et al. 2006, van Raaij et al. 2008, Efe et al. 2011). Reports regarding the influence of gender have also been inconsistent (Aglietti et al. 2003, Fletcher et al. 2006, van Raaij et al. 2008). The reasons for these discrepancies may be differences in study size, loss to follow-up, patient selection, or other factors. In Sweden, the risk of revision after knee arthroplasty has been similar for both sexes (SKAR 2010) and we have no clear explanation for the observed difference after HTO.

Our finding of increased revision risk with age for HTO is opposite to what has been found for both UKA (SKAR 2010, W-Dahl et al. 2010b) and TKA (SKAR 2010), for which there is a marked inverse relationship. We speculate that the reason for this difference may depend on the aim of the treatment and the expectations of the patients. The aim of an HTO is to delay the progress of a disease already started, with the option of later conversion if needed, while an arthroplasty can be viewed as a “definite” treatment of a damaged joint or part thereof. For TKA patients who are not satisfied, the only available offer is “more of the same”; thus, the bar to revise may be high. Young HTO patients can benefit by delaying arthroplasty surgery, as the TKA revision rate is high in younger patients.

In patients younger than 65 years, the CRR at 10 years after UKA is about 16%, and it is 6% for TKA (SKAR 2010), so the CRR of 29% we found for HTO is considerably higher. Considering the possible causes mentioned above, and the fact that the HTO patients were considerably younger at surgery (51 years) than the UKA patients (58 years) and TKA patients (59 years), the revision rate for HTO may be regarded as acceptable.

If it is considered beneficial to avoid insertion of artificial joint implants in younger and/or physically active patients suffering from OA of the knee. HTO can be considered to be a good choice. With 70% survival at 10 years after surgery, HTO managed to markedly delay knee arthroplasty surgery in the majority of patients. However, further studies are needed to monitor still longer-term results, the effect on quality of life, and the outcome for those converted to knee arthroplasty.

## References

[CIT0001] Aglietti P, Buzzi R, Vena LM, Baldini A, Mondaini A (2003). High tibial valgus osteotomy for medial gonarthrosis: a 10- to 21-year study. J Knee Surg.

[CIT0002] Akizuki S, Shibakawa A, Takizawa T, Yamazaki I, Horiuchi H (2008). The long-term outcome of high tibial osteotomy: a ten- to 20-year follow-up. J Bone Joint Surg (Br).

[CIT0003] Coventry MB, Ilstrup DM, Wallrichs SL (1993). Proximal tibial osteotomy. A critical long-term study of eighty-seven cases. J Bone Joint Surg (Am).

[CIT0004] Dorey F, Nasser S, Amstutz H (1993). The need for confidence intervals in the pre¬sentation of orthopaedic data—current concepts review. J Bone Joint Surg (Am).

[CIT0005] Efe T, Ahmed G, Heyse TJ, Boudriot U, Timmesfeld N, Fuchs-Winkelmann S, Ishaque B, Lakemeier S, Schofer MD (2011). Closing-wedge high tibial osteotomy: survival and risk factor analysis at long-term follow up. BMC Musculoskelet Disord.

[CIT0006] Flecher X, Parratte S, Aubaniac JM, Argenson JN (2006). A 12-28-year followup study of closing wedge high tibial osteotomy. Clin Orthop Relat Res.

[CIT0007] Gstottner M, Pedross F, Liebensteiner M, Bach C (2008). Long-term outcome after high tibial osteotomy. Arch Orthop Trauma Surg.

[CIT0008] Knutson K, Lewold S, Robertsson O, Lidgren L (1994). The Swedish knee arthroplasty register. A nation-wide study of 30,003 knees 1976-1992. Acta Orthop Scand.

[CIT0009] Koshino T, Yoshida T, Ara Y, Saito I, Saito T (2004). Fifteen to twenty-eight years’ follow-up results of high tibial valgus osteotomy for osteoarthritic knee. Knee.

[CIT0010] Naudie D, Bourne RB, Rorabeck CH, Bourne TJ (1999). The Install Award. Survivorship of the high tibial valgus osteotomy. A 10- to -22-year followup study. Clin Orthop.

[CIT0011] Odenbring S, Egund N, Knutson K, Lindstrand A, Larsen ST (1990). Revision after osteotomy for gonarthrosis. A 10-19-year follow-up of 314 cases. Acta Orthop Scand.

[CIT0012] Papachristou G, Plessas S, Sourlas J, Levidiotis C, Chronopoulos E, Papachristou C (2006). Deterioration of long-term results following high tibial osteotomy in patients under 60 years of age. Int Orthop.

[CIT0013] Robertsson O, Ranstam J (2003). No bias of ignored bilaterality when analysing the revision risk of knee prostheses: Analysis of a population based sample of 44,590 patients with 55,298 knee prostheses from the national Swedish-Knee Arthroplasty Register. BMC Musculoskeletal Disorders.

[CIT0014] Schallberger A, Jacobi M, Wahl P, Maestretti G, Jakob RP (2011). High tibial valgus osteotomy in unicompartmental medial osteoarthritis of the knee: a retrospective follow-up study over 13-21 years. Knee Surg Sports Traumatol Arthrosc.

[CIT0015] Spahn G, Kirschbaum S, Kahl E (2006). Factors that influence high tibial osteotomy results in patients with medial gonarthritis: a score to predict the results. Osteoarthritis Cartilage.

[CIT0016] Sprenger TR, Doerzbacher JF (2003). Tibial osteotomy for the treatment of varus gonarthrosis. Survival and failure analysis to twenty-two years. J Bone Joint Surg (Am).

[CIT0017] (2010). The Swedish Knee Arthroplasty Register annual report. http://www.knee.se.

[CIT0018] Tjornstrand B, Egund N, Hagstedt BV (1981). Tibial osteotomy in medial gonarthrosis. The importance of over-correction of varus deformity. Arch Orthop Trauma Surg.

[CIT0019] Trieb K, Grohs J, Hanslik-Schnabel B, Stulnig T, Panotopoulos J, Wanivenhaus A (2006). Age predicts outcome of high-tibial osteotomy. Knee Surg Sports Traumatol Arthrosc.

[CIT0020] W-Dahl A, Robertsson O, Lidgren L (2010a). Surgery for knee osteoarthritis in younger patients. Acta Orthop.

[CIT0021] W-Dahl A, Robertsson O, Lidgren L, Miller L, Davidson D, Graves S (2010b). Unicompartmental knee arthroplasty in patients aged less than 65. Acta Orthop.

[CIT0022] van Raaij T, Reijman M, Brouwer RW, Jakma TS, Verhaar JN (2008). Survival of closing-wedge high tibial osteotomy: good outcome in men with low-grade osteoarthritis after 10-16 years. Acta Orthop.

